# Cross-Sectional Study of the Association between a Deepening of the Upper Eyelid Sulcus-Like Appearance and Wide-Open Eyes

**DOI:** 10.1371/journal.pone.0096249

**Published:** 2014-04-29

**Authors:** Shunsuke Nakakura, Etsuko Terao, Nozomi Nagatomi, Naoko Matsuo, Yoshie Shimizu, Hitoshi Tabuchi, Yoshiaki Kiuchi

**Affiliations:** 1 Department of Ophthalmology, Saneikai Tsukazaki Hospital, Himeji, Japan; 2 Department of Ophthalmology and Visual Sciences, Graduate School of Biomedical Sciences, Hiroshima University, Hiroshima, Japan; Bascom Palmer Eye Institute, University of Miami School of Medicine, United States of America

## Abstract

**Background:**

Deepening of the upper eyelid sulcus (DUES) is a common complication of prostaglandin F2α analog treatment, which causes cosmetic problems. However, identifying this condition using photographs is difficult due to such problems as the camera flash effects, blepharoptosis or wide-open eyes.

**Purpose:**

We investigated the association between a DUES-like appearance and wide-open eyes regarding the presence of wide-open eyes as a cause for overestimating the incidence of DUES.

**Subjects and Methods:**

One eye and the forehead in 100 subjects (31 younger subjects, 30 older subjects and 39 patients with blepharoptosis) were evaluated in the present study. Digital photographs of the subjects with natural open and wide-open eyes were taken with a flash. Five signs (a puffy eyelid, the presence/absence of the upper eyelid sulcus (UES), wrinkles on the forehead with natural open eyes and an increase in the number of wrinkles on the forehead and a DUES-like appearance with wide-open eyes) were judged to be negative or positive by three independent observers. Univariate and multivariate logistic regression analyses were performed to determine the independent predictor(s) of a DUES-like appearance with wide-open eyes.

**Results:**

Fourteen subjects (four young, three old and seven subjects with blepharoptosis) were judged to have a DUES-like appearance with wide-open eyes (14%). The only predictive factor was the presence of UES in the patients with natural open eyes (odds ratio = 17.244, 95% confidence interval: 3.447–86.270, P<0.001). Among the 12 UES-positive subjects, six (50%) exhibited a DUES-like appearance with wide-open eyes.

**Conclusions:**

The presence of wide-open eyes can thus cause a DUES-like appearance. Blepharoptosis itself is not a predictive factor; however, care should be taken not to overestimate the incidence of DUES, especially in patients with UES with natural open eyes, as a DUES-like appearance can be caused by wide-open eyes, even in treatment-naïve patients.

**Trial Registration:**

UMIN000010500

## Introduction

Prostaglandin F2α analogs are widely used to treat ocular hypertension and/or glaucoma based on their efficacy in achieving intraocular pressure reduction and good patient compliance due to the once daily dosing schedule. Conjunctival hyperemia, trichiasis and hyperpigmentation of the iris and eyelids are well known side effects of these agents [1]. Recently, a new side effect, termed “deepening of the upper eyelid sulcus (DUES),” has become a subject of discussion [2]–[10]. DUES is also referred to as “sunken eye” [5] or “periorbital fat atrophy” [6] and is thought to be a clinical symptom of prostaglandin-associated periorbitopathy [7], [8]. In clinical practice, however, evaluating this condition is very difficult, as there is no established diagnostic method, and comparing photographs is the only currently recommended clinical assessment [2]–[10].

Previous reports have shown a high incidence (more than 50%) of DUES, especially among patients treated with bimatoprost and travoprost ophthalmic solution [4], [9], [10]. Inoue et al. reported that, in their study, even when patients are treated with latanoprost ophthalmic solution, which has fewer side effects than bimatoprost or travoprost, DUES was judged to be present in 24% of cases objectively and 12% of cases subjectively [9]. Compared to the severe systematic side effects of beta-blockers such as asthma, arrhythmia and bradycardia, the side effects of prostaglandin F2α analogs are primarily local and minor. However, cosmetic problems, such as hyperpigmentation of the eyelid, trichiasis and DUES, can be severe, especially in female subjects, which raises concerns regarding adherence to glaucoma treatment [2], [5]. Therefore, correctly judging whether DUES is present is a very important issue in glaucoma management.

However, in clinical practice, a high incidence of DUES is not observed such as previous reports [4], [9], [10]. Nevertheless, clinicians usually take photographs of patients before and after prescribing prostaglandin F2α analogs in order to diagnose DUES. Therefore, we consider that bias may affect the judgment of whether DUES is present, which may result in overestimation of the incidence of DUES.

We first considered that patients with blepharoptosis sometimes have a deepened upper eyelid sulcus at baseline. Second, we took into account the fact that the appearance of a deepened upper eyelid sulcus can vary based on the brightness of the office or camera flash. Third, we noted that palpebral fissure changes associated with wide-open eyes are also known to affect the depth of the upper eyelid sulcus (UES) in photographs, which may also influence the appearance of the eyes.

To the best of our knowledge, no previous reports have evaluated or considered the effects of wide-open eyes when judging patients for the presence of DUES. Therefore, the primary purpose of this study was to clarify whether the presence of wide-open eyes can cause a DUES-like appearance by investigating the UES appearance in healthy subjects and patients with blepharoptosis. The second purpose was to elucidate the predictive factors of a DUES-like appearance in subjects with wide-open eyes based on observed facial changes.

## Subjects and Methods

This study received approval from the Institutional Review Board of Saneikai Tsukazaki Hospital and was performed according to the tenets of the Declaration of Helsinki. Healthy subjects were recruited from the hospital between April and July 2013. Written informed consent was obtained from each participant prior to enrollment in this study.

All patients underwent an ophthalmic examination, including fundoscopy without pupil dilation. None of the subjects had a previous history of glaucoma treatment or neuro-ophthalmological disease. Patients who had undergone ocular surgery within the past six months were excluded. Healthy subjects were defined as those with an upper eyelid margin-to-corneal light reflex distance (MRD1) of more than 2 mm, while patients with blepharoptosis were defined as those with an MRD1 of <1.5 mm [11].

Finally, one eye and the forehead in 100 subjects (31 younger subjects, 30 older subjects and 39 patients with blepharoptosis) were evaluated in this study.

### Photography

Experienced photographers (S.N. and E.T.) photographed the forehead and eyes under the same room conditions using a digital single-lens camera (EXLIM EX-ZR200, CASIO, Tokyo, Japan) with a flash, and the MRD1 was measured using a millimeter ruler. We believed that the effects of the camera flash may affect the incidence of a DUES-like appearance, as there are differences in past reports, with some authors having obtained photographs without a flash [4], [8], [10], while others obtained photographs with a flash [7], [9]. Therefore, in this study, we obtained all photographs with a flash in order to more strictly assess the signs of DUES.

**Figure 1:**
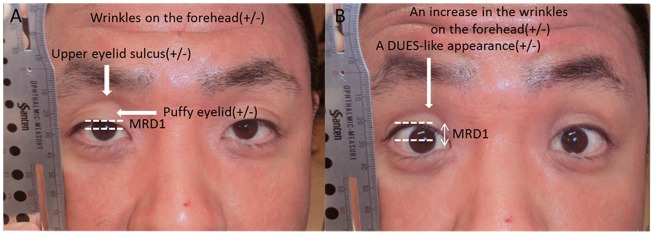
Parameters used in this study. A: Natural open eyes. B: Wide-open eyes. MRD1: margin-to-corneal light reflex distance. A DUES-like appearance: a deepening of the upper eyelid sulcus-like appearance.

First, the patient was asked to relax, and a photograph was taken with natural open eyes. Second, the patient was asked to open their eyes widely (a voluntary eyelid retraction), but not to the maximum, when taking the second photograph (wide-open eyes). The images were printed on glossy paper (EPSON, KL400PSKR) using a digital printer (Colorio me E-350W, EPSON, Tokyo, Japan) and mounted on A4 (210 mm×297 mm)-sized paper in line horizontally for each patient. Among the patients with blepharoptosis in only one eye, the affected eye was selected for the evaluation. Among the healthy subjects and patients with blepharoptosis in both eyes, randomized selection of eyes was performed and measurements of the MRD1 on the two photographs were obtained by E.T in all subjects. The difference between the MRD1 in the patients with natural open and wide-open eyes was defined as the MRD difference, which denoted the degree of opening of the eyes. Investigator-blinded judging was independently performed by three evaluators (S.N, N.N and N.M.), who judged two photographs in each case for the five signs of facial changes (a puffy eyelid, the presence/absence of UES with natural open eyes, the presence/absence of wrinkles on the forehead with natural open eyes, a DUES-like appearance with wide-open eyes and an increase in wrinkles on the forehead with wide-open eyes). Observations unanimously rated positive by the observers were defined as positive. Figure 1 shows the parameters used in this study.

### Statistical analyses

Comparisons of the patient demographics and results of the judgments regarding the five signs between the groups were made using the JMP software program, version 10.0.0 (SAS Institute Inc, Cary, NC, USA) and the Statcel 3 software program (OMS Publishing Ltd., Tokyo, JAPAN). The data are expressed as the mean ± standard deviation (SD). When evaluating differences between the groups, age was analyzed using a one-way ANOVA and Student's *t*-test, while sex, the target eye and judgments regarding the five signs were analyzed using the chi-square test or Fisher's exact probability test and the MRD1 values were analyzed using the nonparametric Kruskal-Wallis test or Mann-Whitney's U test. We applied the Bonferroni correction in cases of multiple comparisons. The degree of agreement among the three observers regarding the five signs was evaluated according to the Fleiss κ factor using the statistical package R, version 2.15.0 (R Foundation for Statistical Computing, Vienna, Austria).

Univariate and multivariate logistic regression analyses were performed to determine whether age, sex, blepharoptosis, a puffy eyelid, UES, wrinkles on the forehead, an increase in wrinkles on the forehead and the MRD difference were independent predictors of a DUES-like appearance in the patients with wide-open eyes using the SPSS ver. 19 software program for Windows. A post–hoc statistical power analysis of the predictive factors was performed using the G*Power 3.1.9 software program (Franz Faul, Kiel University, Kiel, Germany).

P values<0.05 were considered to be statistically significant.

## Results

The patient demographics and measured MRD1 values are shown in Table 1. Statistically significant differences were found between the groups in terms of age, MRD-1 with natural open eyes and MRD1 with wide-open eyes. The Kappa coefficients of the five signs among the three observers are shown in Table 2. A puffy eyelid was the only factor found to be associated with poor agreement (kappa coefficient = 0.237), whereas the other four signs exhibited relatively good agreement (kappa coefficients of 0.464–0.813).

**Table 1:** Patient demographics and measured MRD.

	Younger subjects	Older subjects	Patients with blepharoptosis	p value
Number	31	30	39	
Age (range) (y.o)	27.5±4.3 (22–39)§	67.0±8.9 (53–84)	71.7±8.6 (53–87)	<0.001*
Sex (female)	15	19	20	0.458^†^
Right eye affected	15	17	17	0.558^†^
MRD1 with natural-open eyes (mm)	3.2±1.0	2.7±0.9	0.3±0.4§	<0.001^‡^
MRD1 with wide-open eyes (mm)	5.6±1.4	5.2±1.3	2.4±1.6§	<0.001^‡^
MRD difference (mm)	2.3±1.0	2.5±1.1	2.1±1.4	0.554^‡^

MRD1  =  upper eyelid margin-to-corneal light reflex distance.

MRD difference  =  difference between the MRD1 with natural open and wide-open eyes.

*ANOVA, ^†^chi-square test, ^‡^Kruskal-Wallis test.

§ = statistically significant difference observed compared to the other two groups using Student's *t*-test or the Mann-Whitney U test following the Bonferroni correction.

**Table 2:** Interobserver agreement regarding the five signs.

	Kappa coefficient	Z value	P value
With natural-open eyes			
Puffy eyelid	0.237	4.10	<0.001
Upper eyelid sulcus	0.529	9.17	<0.001
Wrinkles on the forehead	0.813	14.10	<0.001
With wide-open eyes			
An increase in the wrinkles on the forehead	0.607	10.50	<0.001
DUES-like appearance	0.464	8.03	<0.001

**Figure 2:**
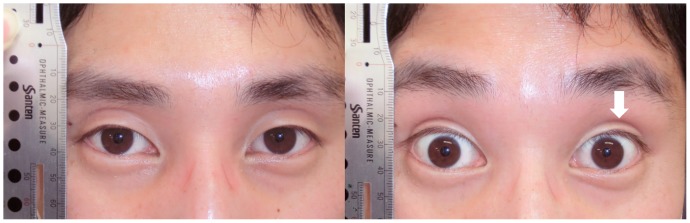
A younger subject judged to have a DUES-like appearance. A healthy 28-year-old male with a DUES-like appearance of the left eye. An UES was judged to be present when he had natural open eyes. All other signs were judged to be negative.

**Figure 3:**
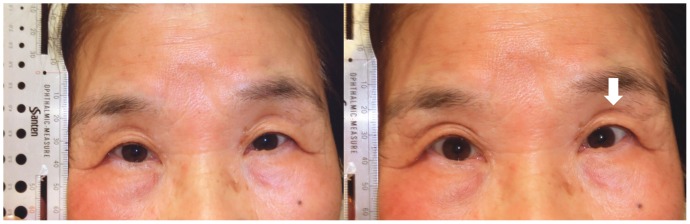
An older subject judged to have a DUES-like appearance. A healthy 72-year-old female with a DUES-like appearance of the left eye. An UES was judged to be present when she had natural open eyes, while other signs were judged to be negative.

**Figure 4:**
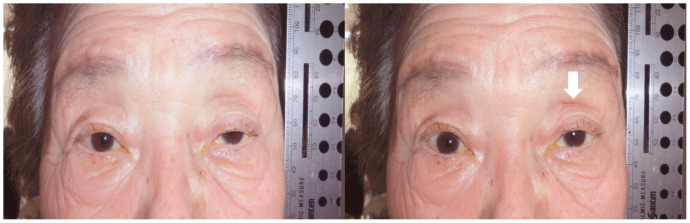
A patient with blepharoptosis judged to have a DUES-like appearance. A 79-year-old female with blepharoptosis of the left eye. A DUES-like appearance, UES and wrinkles on the forehead were all judged to be positive.

The results of the comparisons between the groups with regard to the five signs are shown Table 3. Fourteen subjects (four younger subjects, three older subjects and seven subjects with blepharoptosis) were judged to have a DUES-like appearance with wide-open eyes (14%). The only significant difference between the groups was the presence of wrinkles on the forehead with natural open eyes. Figures 2–4 show the results of the five signs for the DUES-like appearance-positive patients in each group. All study patients in Figures 1 to 4 provided their written informed consent, as outlined in the PLOS ONE consent form, prior to publication of their photographs.

**Table 3:** Prevalence of the five signs among the three groups.

	Younger subjects (N = 31)	Older subjects (N = 30)	Patients with blepharoptosis (N = 39)	p value
With natural-open eyes				
Puffy eyelid (+:−)	6∶25	1∶29	9∶30	0.071
Upper eyelid sulcus (+:−)	2∶29	6∶24	4∶35	0.242
Wrinkles on the forehead (+:−)	1∶30*	13∶17	28∶11	<0.001
With wide-open eyes				
An increase in the wrinkles on the forehead (+:−)	11∶20	12∶18	7∶32	0.102
DUES-like appearance (+:−)	4∶27	3∶27	7∶32	0.627

All p values were obtained using the chi-square test.

* = statistically significant difference observed compared to the other two groups using Fisher's exact probability test and the Bonferroni correction.

### Univariate and multivariate logistic regression analyses of independent predictors of a DUES-like appearance

Table 4 shows the results of the univariate and multivariate logistic regression analyses.

**Table 4:** Results of the univariate and multivariate logistic regression analyses of the independent predictors of a DUES-like appearance.

	DUES-like appearance (-) (n = 86)	DUES-like appearance (+) (n = 14)	Univariable analysis			Multiple logistic regression analysis		
			OR	95%CI	P value	OR	95%CI	P value
Age (y.o) (per 10 years)	56.1±21.3	59.6±20.5	1.087	0.821,1.438	0.561	0.905	0.571,1.436	0.672
Male/female	46/40	8/6	1.159	0.371,3.636	0.799	1.215	0.311,4.744	0.779
Blepharoptosis (+/−)	32/54	7/7	1.687	0.542,5.252	0.366	4.547	0.795,26.000	0.089
Puffy eyelid (+/−)	16/70	0/14	-					
**Upper eyelid sulcus (+/−)**	**6/80**	**6/8**	**10.000**	**2.605,38.382**	**<0.001**	**17.244**	**3.447,86.270**	**<0.001**
Wrinkles on the forehead (+/−)	36/50	6/8	1.042	0.332,3.264	0.944	0.492	0.101,2.405	0.381
An increase in the wrinkles on the forehead (+/−)	25/61	5/9	1.356	0.413,4.448	0.616	2.001	0.467,8.568	0.350
MRD difference (mm) (per 1 mm)	2.3±1.3	2.0±0.9	0.837	0.523,1.339	0.457	0.942	0.537,1.652	0.835

OR = odds ratio, CI = confidence interval.

UES was found to be the only significant predictive factor of a DUES-like appearance in both the univariate (odds ratio (OR) = 10.000, 95% CI: 2.605–38.382, P<0.001) and multivariate logistic regression (OR = 17.244, 95% CI: 3.447–86.270, P<0.001) analyses. Blepharoptosis (OR = 4.547, 95% CI: 0.795–26.000, P = 0.089) and the MRD difference (OR = 0.942, 95% CI: 0.537–1.652, P = 0.835) were not found to be predictive factors in the multivariate logistic regression analysis.

The post-hoc statistical power analysis showed that UES had a statistical power of 0.902 in the univariate analysis, while the other covariates exhibited a statistical power of 0.847 and an R^2^ of 0.179 in the multivariate logistic regression analysis.

Table 5 shows the incidence of a DUES like-appearance based on the various predictive factors.

**Table 5:** Incidence of a DUES like-appearance according to predictive factors.

		Overall	DUES-like appearance (-)	DUES-like appearance (+)	Rate of DUES- like appearance (%)
Sex	male	54	46	8	14.8
	female	46	40	6	13.0
Blepharoptosis	+	39	32	7	17.9
	-	61	54	7	11.5
Puffy eyelid	+	16	16	0	0
	-	84	70	14	20.0
**Upper eyelid sulcus**	**+**	**12**	**6**	**6**	**50.0**
	-	88	80	8	9.1
Wrinkles on the forehead	+	42	36	6	14.3
	-	58	50	8	13.8
An increase in the wrinkles on the forehead	+	30	25	5	16.7
	-	70	61	9	14.8

Among the 12 UES-positive subjects with natural open eyes, six (50%) had a DUES like-appearance with wide-open eyes. However, the incidence of a DUES like-appearance with respect to the other parameters was relatively low (0% to 20%), and subjects with a puffy eyelid were especially resistant to having a DUES like-appearance with wide-open eyes.

## Discussion

In this study, we showed that there is an association between a DUES-like appearance and wide-open eyes and found that the presence of UES at baseline is a factor predicting the development of a DUES-like appearance with wide-open eyes based on a multivariate logistic regression analysis. In detail, among the 12 subjects with UES with natural open eyes, six (50%) developed a DUES-like appearance with wide-open eyes. On the other hand, among the 88 subjects without UES, nine (9.1%) developed a DUES-like appearance with wide-open eyes. These results indicate the possibility that the incidence of DUES may be overestimated in patients with an UES with natural open eyes, if such patients open their eyes widely for the camera, and that the deepened UES observed in these subjects is not caused by treatment with prostaglandin F2α analogs. Our results also suggest the possibility that previous reports overestimated the incidence of DUES [4], [9], [10]. According to previous reports, in comparisons between subjective evaluations based on photographs and objective evaluations using questionnaires, the presence of DUES was less frequently reported in the subjective evaluations than in the objective evaluations. Therefore, when judging whether a glaucoma patient has DUES using an objective method employing photography, care should be taken in patients with wide-open eyes not to overestimate the presence of DUES.

**Figure 5:**
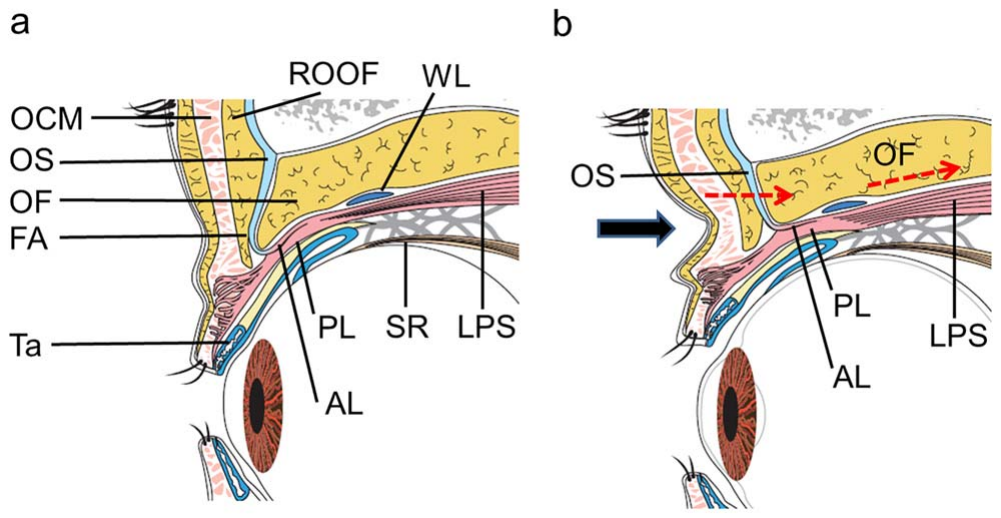
A schematic drawing of the upper eyelid and a possible mechanism underlying the presence of a DUES-like appearance. a. A schematic drawing of the upper eyelid associated with natural open eyes. b. A schematic drawing of the upper eyelid associated with wide-open eyes. A DUES-like appearance occurs when LA draws OS and fat toward the back of the eyeball. LPS: levator palpebrae superioris muscle, WL: Whitnall ligament, OF: orbital fat, OS: orbital septum, AL: anterior layer of LA, PL: posterior layer of LA, FA: fibroadipose layer, OCM: orbicularis oculi muscle, ROOF: retro-orbicularis oculi fat; SR: superior rectus muscle. The black arrow indicates the DUES-like appearance. The red dotted arrows show the effects of the LPS and LA.

The causes of UES include congenital factors (i.e., among the younger subjects in the present study) and aging [12], and UES was observed at a similar rate in each group (P = 0.242) in this study. Owing to the displacement of periorbital fat and subcutaneous tissue that occurs with aging, the bony architecture of the orbit becomes more evident, making the globe appear sunken, with advancing age [12]. We speculate that the mechanisms underlying the presence of a DUES-like appearance in patients with wide-open eyes include the following: the levator aponeurosis (LA) connecting the constricted levator palpebrae superioris muscle draws the orbital fat and orbital septum (OS) toward the back of the eyeball, thus increasing the depth of the UES (Figure 5). This phenomenon occurs because LA originates from the levator palpebrae superioris muscle, consisting of anterior and posterior layers, including smooth muscle [13]. The anterior layer is comprised of thick robust fibrous tissue and ends in the junctional region with OS and submuscular fibroadipose tissue, enabling it to directly pull the preaponeurotic fat pad in cooperation with OS and submuscular fibroadipose tissue [13]. These speculations may be confirmed by orbital magnetic resonance imaging (MRI) using specific coils [14]. The use of such custom-designed coils allows for the visualization of eyelid structures, such as OS, LA and orbital septa. However, in order to adequately evaluate the eyelid and sulcus, it is necessary to examine the patient while in the sitting or upright position, and most MRI examinations can only be performed with the subject in the supine position.

The MRD differences among the three groups were not statistically significant, possibly because the subjects were asked to open their eyes widely, but not to the maximum degree. The levator function is generally decreased in subjects with blepharoptosis; however, we assumed prior to conducting this study that no subjects would initially try to open their eyes to the maximum level when in front of a camera.

Wrinkles on the forehead in subjects with natural open eyes were observed significantly more often among the patients with blepharoptosis than in those without. It is likely that the consistent strain on the frontal belly of the occipitofrontalis muscle due to the maintenance of wide-open eyes in daily life leads to the formation of wrinkles on the forehead in patients with blepharoptosis. In the current study, the presence of blepharoptosis and the MRD difference were not found to be predictive factors for a DUES-like appearance. We suspect that the reason for this finding is that Asian patients tend to have thick eyelids and a full-appearing UES compared to Caucasian patients [15], thus a deepened UES associated with blepharoptosis and/or the degree of opening of the eyes are relatively less prominent in Asian individuals, although LA of the upper eyelid draws the orbital fat and OS back toward the eyeball.

Our results therefore suggest the possibility that the incidence of DUES is more frequently overestimated in Caucasian patients than in Asian patients, as Asian patients have thicker eyelids and a more full-appearing UES [15]. Previous prospective reports regarding the incidence of DUES have almost been from Japan [4], [8]–[10]; therefore, further investigations of the incidence of DUES in Caucasian countries may bring about interesting discussions regarding ethnicity differences.

Glaucoma is a type of optic neuropathy characterized by specific and progressive damage to retinal ganglion cells. Decreasing the intraocular pressure efficiently prevents the progression of glaucoma in the visual field, even among patients with normal tension glaucoma [16]. Therefore, glaucoma is a medical and, ultimately surgical, issue. Various topical anti-glaucoma eye drops are used in the clinical setting, although they have various direct actions and side effects. The ocular side effects of prostaglandin F2α analogs differ based on the ophthalmic solution and the methods used in previous reports [1]. Such complications include conjunctival hyperemia (14.8%–68.6%), eyelash growth (4.4%–57.1%) and skin discoloration (1.5%–2.9%). Upper respiratory tract infection is the most common systemic adverse effect of prostaglandin F2α analogs (occurring in approximately 4% of patients) [1]. On the other hand, the most common ocular side effects of beta-blocker ophthalmic solutions (conjunctival hyperemia, superficial punctate keratitis, etc.) occur in approximately 22.5% of cases (123/547 subjects). Meanwhile, systemic side effects of beta-blocker ophthalmic solutions include central nervous system effects, occurring in 14.4% of cases (79/547 subjects)(depression, anxiety, confusion, etc.), cardiac issues, occurring in 12.8% of cases (70/547 subjects)(bradycardia, arrhythmia, heart failure, etc.), and pulmonary complications, occurring in 6.8% of cases (37/547 subjects)(dyspnea, airway obstruction, pulmonary failure) [17]. The most common ocular side effects of topical carbonic anhydrase inhibitors include blurred vision, tearing and dryness, being observed in approximately 1%-5% of patients. The most common systemic side effect of one topical carbonic anhydrase inhibitor (dorzolamide 2%) has been reported to be a bitter or metallic taste, reported in 27% of patients [18].

The incidence of DUES (8.0%–60% objectively) reported in previous studies [4], [9], [10] is relatively high compared to the incidence of other side effects of prostaglandin F2α analogs, beta-blockers and carbonic anhydrase inhibitors. Therefore, although DUES may not be life-threatening and is limited to treatment with F2α analogs, clinicians should carefully watch patients for the development of DUES, as it is a major cosmetic problem in those with glaucoma and can decrease compliance with treatment.

One limitation of this study is that the agreement (κ) regarding the presence of a puffy eyelid among the three observers was low, at 0.237. Asian eyelids are thicker than occidental eyelids, as Asian patients possess a marked excess of pretarsal tissue and often exhibit pseudoblepharoptosis and epiblepharon [15]. Therefore, judging whether a patient has a puffy eyelid is difficult because no standard has been clearly defined. The second limitation of this study is the strict definition of blepharoptosis (a MRD1 of 1.5 mm or less), as a large number of Asian individuals without any subjective symptoms [19] would be classified as having blepharoptosis if the Caucasian criterion of an MRD1 >2.8 mm was used [20]. The third limitation is that we did not perform a sample size estimation prior to sampling. Therefore, we used a post-hoc statistical analysis to determine whether the only predictive factor (UES with natural open eyes) fulfilled the statistical power requirements. The results showed a statistical power of 0.902 in the univariate analysis, while other covariates demonstrated a statistical power of 0.847 and R^2^ of 0.179 in the multivariate logistic regression analysis.

In conclusion, we herein showed that there is an association between a DUES-like appearance and wide-open eyes. This finding indicates that clinicians should take care not to misjudge the presence of DUES when evaluating glaucoma patients, especially those with UES with natural open eyes.
